# Understanding knowledge, perception, attitude and behaviour on sustainable healthcare waste management practices in selected National Health Service Trusts in the Northwest of England

**DOI:** 10.1177/0734242X251374491

**Published:** 2025-10-04

**Authors:** Timothy Kurannen Baaki, Christaline Wijekoon, Ibijoke Idowu, Lakna Ganegodage

**Affiliations:** Faculty of Engineering and Technology, Liverpool John Moores University, Liverpool, UK

**Keywords:** Attitude, healthcare waste, knowledge, NHS, perception, sustainable healthcare waste management, training, United Kingdom

## Abstract

Of the carbon emissions that the National Health Service (NHS) directly produces, waste and water currently make up about 21%, only second to building energy. This study evaluated current knowledge, perception, attitude and behaviour on sustainable healthcare waste management (SHCWM) practices within selected NHS Trusts in Northwest England post-COVID-19 as part of wider research to develop a resilient assessment tool for SHCWM implementation within NHS Trusts in the United Kingdom. The study utilised a questionnaire survey of hospital staff across three NHS Trusts. A total of 58 respondents from clinical and non-clinical roles were analysed. The results showed lack of periodic training, reflected in generally low levels of knowledge on health/environmental risks of healthcare waste management (HCWM), SHCWM practices and legislation on HCWM in the United Kingdom and deficiencies in segregation practice. Statistical tests showed significant differences in attitude among age groups and roles. Those under the age of 35 demonstrated more positive attitude towards SHCWM, whereas clinical support staff demonstrated more positive attitude towards SHCWM compared to doctors and nurses. While the findings demonstrate a lack of periodic training on SHCWM, there appears to be a generally positive perception and attitude towards SHCWM practices. Where SHCWM initiatives are introduced, the findings suggest that staff would be willing to engage and participate. This paper gives those leading sustainability efforts at NHS Trusts a snapshot of current sentiment towards SHCWM and elevates the need to develop minimum mandatory periodic training to improve staff knowledge and practice on SHCWM as part of efforts towards a net zero future.

## Introduction and background

As one of the largest public sector organisations in the world, the United Kingdom National Health Service (NHS), responsible for public healthcare provision in the United Kingdom, generates substantial amounts of waste that significantly contributes to emissions in the United Kingdom and by implication, globally. It has been reported that, public healthcare provision in the United Kingdom is responsible for more than a third (40%) of UK public sector emissions (NHS England, 2021) and contributes 4% to the total carbon footprint of the United Kingdom (NHS England, 2022a). Waste and water contribute about 4% to the NHS’s total carbon emission. However, of the carbon emissions that the NHS directly produces, waste and water currently make up about 21%, only second to building energy (NHS England, 2022a). Waste generation within the NHS is estimated at 3% annual growth rate (NHS England, 2023). Recent figures have reported clinical waste alone to be up to 156,000 tonnes a year (NHS England, 2023). The Royal College of Nursing (RCN) (2018) reported that participating NHS Trusts, representing 69% of all NHS Trusts, in a freedom of information request reported generating a total 185,233 tonnes of waste in 2015/2016. Of that amount, municipal waste accounted for 59% (110,103 tonnes), infectious waste 33% (60,700 tonnes) and offensive waste 8% (14,350 tonnes). In 2022, the NHS, through the Health and Care Act 2022, became the first public sector organisation worldwide to set binding carbon emissions targets to achieve net zero carbon emissions by 2040 for directly controlled emissions and by 2045 for indirect emissions (NHS England, 2022a). There are growing expectations for healthcare organisations to introduce sustainable approaches to healthcare waste management (HCWM), which on average accounts for 5% of carbon footprint in OECD countries, China and India, comparing, in significance, to the food sector (Pichler et al., 2019). While progress has been reported (Cohen and Howard, 2015; Practice Greenhealth, 2014; Voudrias, 2018), these expectations have, arguably, become even more pertinent considering disruptions brought about by the COVID-19 pandemic. There is recognition of HCWM as a significant component of sustainability within healthcare settings (Azmal et al., 2014). In 2023, the NHS published the *Clinical Waste Strategy* in order to set a strategy towards a more sustainably approach to clinical waste management (NHS England, 2023). Also, the *Health Technical Memorandum 07-01: Safe and sustainable management of healthcare waste* guidance has been revised to include specific guidance on sustainable healthcare waste management (SHCWM) (NHS England, 2022b). SHCWM refer to a range of deliberate sustainable practices across the HCWM chain that aims to reduce carbon footprint and negative impact of healthcare waste on human health and the environment. These broadly include waste prevention and minimisation through for example green purchasing, effective segregation; reuse and recycling; and use of environmentally friendly options to treat and dispose of wastes arising from healthcare provision without compromising patient and staff safety. Healthcare waste (HCW) on the other hand is ‘all waste’ produced by or associated with healthcare provisions, including research establishments, home treatments, etc. (Chartier et al., 2014; Thakur and Ramesh, 2015). The majority of HCW is non-risk, ranging between 75% and 90% of the total waste stream (Chartier et al., 2014), with non-risk or domestic-type having potential for reuse and recycling (Marmot, 2010; Scally, 2009; Voudrias, 2018). Domestic-type or general HCW comprises components such as plastics, paper, wood, cardboard, glass and food wastes and do not carry hazardous or infectious risks in their nature but has significant negative impact on the environment if inappropriately disposed (Wilburn, 2012). The NHS for example has historically spent tens of millions disposing of waste that could by diverted, with Hutchins and White (2009) reporting up to £73 million in waste disposal costs in 2005. The RCN (2018) reported that appropriately reclassifying 20% of infectious waste to municipal waste and 50% of infectious waste to offensive waste could save the NHS a total of about £7.718 million per annum. The NHS *Clinical Waste Strategy* if well implemented estimates more than £11 million per year in costs savings (NHS England, 2023), suggesting the NHS still incurs high disposal costs for healthcare waste. Following the COVID-19 pandemic, studies reported significant increase in healthcare waste generation (Peng et al., 2020) and disruptions in sustainable approaches to HCWM such as rampant purchasing of personal protective equipment (PPE) that ended up unused (Iacobucci, 2024), and no or limited segregation practice on the assumption that all HCW was potentially contaminated by the COVID-19 virus (Maalouf and Maalouf, 2021). As segregation is one, if not the most essential aspect of the HCWM process, improper or inadequate segregation practices can significantly impact the waste management chain such as determining suitable treatment and disposal options (Barbosa and Mol, 2018; Mmereki et al., 2017) and implementing sustainable practices such as reuse and recycling. Although majority of HCW is non-risk with potential for reuse and recycling, it is important that those generating, handling and managing HCW understand the full range of health and environmental risks associated with HCWM as well as safe and sustainable practices. Following the COVID-19 pandemic, a number of studies around the world have evaluated HCWM generally, addressing elements such as knowledge, attitude and practices (KAP) of healthcare professionals towards COVID-19 waste (Shekoohiyan et al., 2022; Thilagavathi et al., 2021), challenges of managing and handling medical waste during the pandemic (Manupati et al., 2021; Mawkhlieng and Majumdar, 2021), waste generation during COVID (Al-Omran et al., 2021; Chowdhury et al., 2022; Dehal et al., 2022; Garlasco et al., 2022; Martins et al., 2021; Singh et al., 2022; Tsai, 2021). It is evident that studies have been conducted addressing aspects such as waste generation, knowledge, attitude and practices, and challenges of HCWM, however, few have focused on SHCWM. Thakur (2021) developed a framework for PESTEL dimensions of SHCWM, identifying political, legal and environmental issues as immediate policy concerns. Zhu et al. (2022) proposed an approach to repurpose disposable nitrile rubber gloves into sustainable road material as a sustainable method to combat pandemic waste. Torkayesh et al. (2021) proposed a multi-objective optimisation model for HCWM focusing on minimisation of transportation, processing and establishment costs, minimisation of environmental risks and emissions related to transportation, and maximising job creation opportunities. Studies have highlighted the role of training, knowledge, risk perception, attitude and behaviour change in advancing safe HCWM (Baaki et al., 2022; Botelho, 2012; de Aguiar Hugo and Lima, 2021; El-Gilany et al., 2017; Fraifeld et al., 2021; Mathur et al., 2011; Ozder et al., 2013; Shaheen et al., 2022; Wu et al., 2020) and SHCWM implementation (Baaki, 2019; Tudor et al., 2007). Baaki (2019) in a study of hospital staff in two teaching hospitals in Malaysia found training and education was a critical success factor for successful SHCWM implementation. A cross-sectional study by Mathur et al. (2011) found that doctors, nurses and laboratory technicians exhibited better knowledge on biomedical waste management generally compared to sanitary staff, however nurses and laboratory technicians exhibited better knowledge regarding specific aspects such as colour coding and waste segregation. The study also reported that sanitary staff exhibited poor practice compared to doctors, nurses and laboratory technicians. Other studies have reported improvements in knowledge and practice following training interventions (Fraifeld et al., 2021; Kumar et al., 2015). HCWM approaches and practices are directly linked to how HCW is perceived, which impacts HCWM processes, in particular segregation. Janmaimool et al. (2024) reported that perceived negative health impacts of healthcare waste directly influenced infectious waste minimisation and infectious waste segregation, whereas perceived negative environmental impacts affected infectious waste collection awareness. Ferreira and Teixeira (2010) in a study in Portugal found that there was higher risk perception of healthcare waste associated with the environment and those handling waste, linked to level of knowledge. Despite legislation and policies in place, a change in perception and behaviour towards sustainable practices have been reported as important factors towards sustainability practices (Baaki, 2019; DEFRA, 2005; Tudor et al., 2007). Behaviour change, for example, is important, as demonstrated by Tudor et al. (2007), who found intended behaviour did not match actual behaviour with clinical waste bins containing high quantities of wastes considered domestic-type waste. However, few studies have specifically addressed training, knowledge, attitudes and behaviour on SHCWM practices in the United Kingdom, and there is none to mind that has particularly addressed training, knowledge, perception, attitudes and behaviour on SHCWM practices in the United Kingdom following on from the COVID-19 pandemic. The research by Tudor et al. (2007) is one notable study that has previously investigated links between intentions and SHCWM in the United Kingdom. In their study investigating perceived link between management of wastes and spread of infections, Tudor et al. (2010) highlighted the importance of further investigations into perceptions and beliefs of staff towards HCWM. Perception is how organisms interpret and organise sensation to create a meaningful experience of the world (Pickens, 2005). In this study, perception refers to views and feelings about the nature of HCW borne out of experience and perceived norms such as feelings about the risk/hazardous nature of HCW. Perception and attitude are closely related (Kersten and Yuille, 2003; Pickens, 2005). Attitude refers to a person’s mindset, disposition or tendency to behave in a particular way that comes from both their temperament and experience (Pickens, 2005). A person’s attitude towards SHCWM, for example, shows their viewpoint, feelings and likely behaviour towards SHCWM practices. Behaviour is construed to include intended and actual behaviour, which are significantly linked (Ajzen, 1991). The basis for assessing knowledge, perceptions, attitudes and behaviour in this study integrates principles form the theory of planned behaviour (Ajzen, 1991; Conner and Armitage, 1998), and the knowledge, attitudes, practices model (Andrade et al., 2020; Lam et al., 2024). The theory of planned behaviour which is an extension of the theory of reasoned action assumes that intention to perform specific behaviour is influenced by attitude, subjective norm and perceived behavioural control. Both theories have provided a broad framework for investigating intentions and behaviours in a wide range of research areas on sustainability (Chen, 2016; Yadav and Pathak, 2016) including waste management research (Ghani et al., 2013; Tonglet et al., 2004) and in particular, HCWM (Tudor et al., 2007). The main objective of this study was to evaluate current knowledge, perception, attitude and behaviour on SHCWM practices in selected NHS Trusts in Northwest England. The main objective was further divided into three specific objectives namely, to (a) examine the training regime on HCWM within NHS Trusts, (b) evaluate staff knowledge on SHCWM practices and legislation and (c) evaluate perception, attitude and behaviour of staff towards SHCWM. This study is part of a wider research study to develop a resilient assessment tool for SHCWM implementation within NHS Trusts in the United Kingdom.

## Materials and methods

This study was carried out in three NHS Trusts in the Northwest of England. One of the Trusts had three separate hospital sites so in total, five hospital sites were evaluated. One of the NHS Trusts was a general Trust providing emergency, maternity, paediatric care with approximately 700 beds, whereas 2 were specialist Trusts, 1 with approximately 100 beds and 1 with approximately 150 beds. At all the observed Trusts, HCWM was under the responsibility of the Estates and Facilities with only one of the Trusts having a designated waste manager. The study utilised questionnaire survey of staff responsible for generating and managing waste within the hospitals. The inclusion criteria therefore covered staff who were 18 years and above who were directly generating and handling waste, those responsible for establishing waste policy and practice either at hospital or department level. Those in purely waste handling or custodial roles such as domestic housekeeping were excluded from the study. Following Krejcie and Morgan (1970), a sample size of 357 was deemed appropriate for the population. As the three Trusts offered distinct services, stratified random sampling was used to survey the respondents in attempt to have representative samples for the three Trusts. The self-administered online survey questionnaire was developed using the JISC Online Survey tool and was divided into two sections and utilised principles from the KAP model for assessing KAP (Andrade et al., 2020; Lam et al., 2024). The first section included demographic information of the respondents and general information about the nature of waste they generated. The second section included questions on the level of training, knowledge, perception, attitude and behaviour of staff towards sustainable HCWM practices. The questions on training required the respondents to indicate how often they received periodic training on health/environmental risks of HCWM and SHCWM practices with the following options: no training, once a year, twice a year, three times a year, four times a year, five or more times a year. The questions on knowledge required a self-assessment of level of knowledge based on a description of a range of health/environmental risks of HCWM, SHCWM practices and legislation on HCWM in the United Kingdom. Although a subjective measure, self-reported measures are widely used in assessing level of knowledge (Donegan et al., 2025; Olaifa et al., 2018). The respondents were required to rate their level of knowledge on a scale of 0–5 (0 = knowledge; 5 = very high knowledge). The questions on perception, attitude and behaviour required respondents to demonstrate their agreement or not on aspects that assessed their risk perception, attitude and behaviour (intended and self-reported) generally on a dichotomous scale (yes/no) and Likert scale (strongly agree to strongly disagree). Example of yes/no questions included: (a) Do you consider all waste generated in your hospital or department potentially infectious or hazardous? (b) Would you be willing to reuse devices and equipment if they were deemed safe? Example of Likert scale questions included: (a) Segregation at source of waste generation reduces volume of hazardous/infectious waste; (b) I think carefully about the waste type before I discard waste items in the available bins. Prior to distributing the questionnaire, face validity of the questionnaire was ethe survey questionnaire was piloted first for face validity with three people with HCWM experience. The questionnaire was improved and further piloted among 20 staff at one of the observed trusts. The questionnaire was further improved for conciseness and clarity. The data obtained from the pilot was used to perform a reliability analysis, which returned a Cronbach’s alpha (α) of 0.744 for behaviour and 0.827 for attitude. The online questionnaire was distributed to 360 respondents via official communication links at the respective observed NHS Trusts in February 2024, and the respondents were given 2 weeks to respond. After successive follow-ups over a period of 2 months, 61 questionnaires were returned. After scrutinising the returned questionnaires, three were found to be incomplete and discarded. A total of 58 questionnaires were valid for analysis, yielding a valid response rate of 16.1%. The International Business Machines Corporation (IBM) IBM Statistical Packages for Social Sciences (SPSS) software v29.0.2.0 was used to analyse the questionnaire responses using frequency distribution. Kruskal–Wallis and Mann–Whitney *U*-tests were used considering that small sample and data was adjudged not to be normally distributed (Field, 2009) to evaluate differences in knowledge, perception, attitude and behaviour between various demographic groups. Statistical significance was determined at a *p*-value of 0.05 and 95% confidence interval.

## Results

### Profile of survey respondents

As shown in Table 1, majority of respondents were between the ages of 34 and 54 (58.6%), followed by those 18 and 34 (22.4%), then those over 55 (19%). Gender, skewed more towards females with 63.8% of the respondents female and 36.2% male. With respect to day-to-day roles, majority (44.8%) were in administrative roles, followed by (36.2%) in clinical support. Doctors and nurses were 10.3% and 8.6% of the respondents, respectively. Majority of the respondents had 6 years or more experience working in their current department, followed by those with 3–5 years of experience (36.2). Those with 1 year or less experience in their current department were 12.1%, with those with 4–5 years of experience 8.6%. This shows that approximately 88% of the respondents had 2 years or more experience working in their current department and their experience of activities within their respective departments did not span only a short period of time.

**Table 1.** Profile of survey respondents (*n* = 58).

Item	Description	Frequency	Percentage
Age	18–34	13	22.4
	35–54	34	58.6
	55 and above	11	19
	Total	58	100
Gender	Female	37	63.8
	Male	21	36.2
	Total	58	100
Years of working in current department	1 year or less	7	12.1
	2–3 years	21	36.2
	4–5 years	5	8.6
	6 years and above	25	43.1
	Total	58	100
Designation	Doctor	6	10.3
	Nurse	5	8.6
	Admin	26	44.8
	Clinical support	21	36.2
	Total	58	100

### Waste generation and segregation

**Figure 1.**
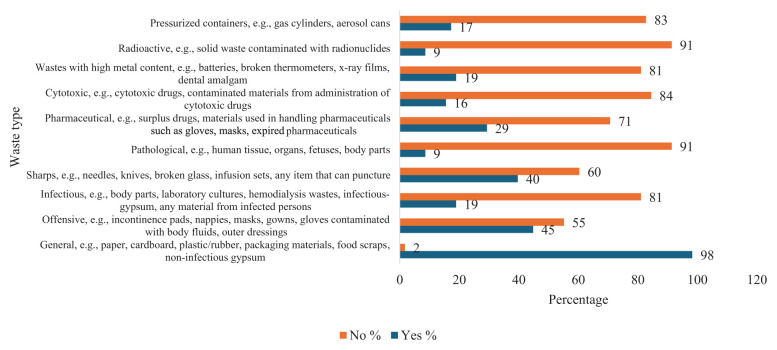
Type of waste generated.

The respondents were asked to indicate the type of waste they generated. The question was based on their own experience of waste generation. As shown in Figure 1, 98% of the respondents indicated they generated general waste followed by 45% offensive waste, 40% sharps waste, 28% pharmaceutical waste, 19% each for infectious waste and wastes with high metal content, 17% pressurised containers and 16% cytotoxic waste. Only 9% indicated they generated pathological waste and radioactive waste, respectively. The results indicate that the full spectrum of HCW streams were generated by the respondents, with almost all the respondents generating general waste.

To evaluate the level of segregation practice and compliance, the respondents were asked to identify the appropriate colour code for the different types of HCW streams and to indicate where they put each type of waste once they generated it. This was based on the colour code and bin type stipulated in the *Health Technical Memorandum 07-01: Safe and sustainable management of healthcare waste*. In Table 2, the results show that 42 respondents chose the correct colour code for general waste, whereas 8 respondents chose the wrong colour code.

**Table 2.** Waste segregation practice colour code.

Waste type	Colour code							
	Black	Yellow/black	Yellow	Orange	Purple	Red	Blue	White
General waste	42	3	0	1	0	0	1	3
Offensive waste	0	19	6	3	0	0	0	0
Infectious waste	0	3	6	8	0	1	0	0
Sharps waste	1	3	4	2	0	0	0	0
Pathological	0	1	2	1	0	2	0	0
Pharmaceutical waste	1	3	1	2	0	0	2	0
Cytotoxic waste	0	0	0	0	3	0	0	0
Wastes with high metal content	1	0	0	1	0	0	0	2
Radioactive waste	0	1	3	0	0	0	0	1
Pressurised containers	1	0	0	1	0	0	0	0

For offensive waste, 19 respondents chose the correct colour code, whereas 9 respondents chose the wrong colour code. For infectious waste, nine respondents chose the correct colour code, whereas nine respondents chose the wrong colour code. For sharps waste, six respondents chose the correct colour code, whereas four respondents chose the wrong colour code. For pathological waste, five respondents chose the correct colour code, whereas one respondent chose the wrong colour code. For pharmaceutical waste, two respondents chose the correct colour code, whereas seven respondents chose the wrong colour code. For cytotoxic waste, three respondents chose the correct colour code, whereas zero respondent chose the wrong colour code. For wastes with high metal content, two respondents chose the correct colour code, whereas two respondents chose the wrong colour code. For radioactive waste, three respondents chose the correct colour code, whereas two respondents chose the wrong colour code. For pressurised containers, one respondent chose the correct colour code, whereas one respondent chose the wrong colour code. The results in Table 3 showed that for general waste, 31 respondents indicated they used the correct waste bin, whereas 1 respondent indicated they used the wrong waste bin. For offensive waste, 11 respondents indicated they used the correct waste bin, whereas 2 respondents indicated they used the wrong waste bin. For infectious waste, six respondents indicated they used the correct waste bin, whereas three respondents indicated they used the wrong waste bin. For sharps waste, 23 respondents indicated they used the correct waste bin, whereas 4 respondents indicated they used the wrong waste bin. For pathological waste, two respondents indicated they used the correct waste bin, whereas three respondents indicated they used the wrong waste bin. For pharmaceutical waste, five respondents indicated they used the correct waste bin, whereas nine respondents indicated they used the wrong waste bin. For cytotoxic waste, four respondents indicated they used the correct waste bin, whereas seven respondents indicated they used the wrong waste bin. For wastes with high metal content, 4 respondents indicated they used the correct waste bin, whereas 10 respondents indicated they used the wrong waste bin. For radioactive waste, nine respondents indicated they used the correct waste bin, whereas five respondents indicated they used the wrong waste bin. For pressurised containers, five respondents indicated they used the correct waste bin, whereas four respondents indicated they used the wrong waste bin. The results indicate some instances of identifying wrong colour codes and disposing of wastes in the wrong bin potentially resulting in comixing of hazardous HCW waste general HCW.

**Table 3.** Waste segregation practice.

Waste type	Waste receptacle or bin												
	Dedicated recycle/recovery bin	Black bag	Yellow/black stripped bag	Red leak-proof lidded rigid bin	Orange leak-proof lidded rigid bin	Purple leak-proof lidded rigid bin	Blue leak-proof lidded rigid bin	Orange leak-proof lidded sharps container	Yellow leak-proof lidded sharps container	Purple leak-proof lidded sharps container	Yellow bag with radioactive symbol	Lead foil	Leak-proof lidded rigid container with mercury suppressant
General waste	10	21	0	0	0	0	1	0	0	0	0	0	0
Offensive waste	0	0	11	0	1	0	1	0	0	0	0	0	0
Infectious waste	0	0	2	2	4	1	0	0	0	0	0	0	0
Sharps waste	1	0	0	0	2	0	0	5	17	1	1	0	0
Pathological	1	0	1	1	1	0	0	1	0	0	0	0	0
Pharmaceutical waste	2	0	2	0	1	2	3	2	1	1	0	0	0
Cytotoxic waste	1	0	2	0	1	4	1	0	1	1	0	0	0
Wastes with high metal content	8	1	0	0	0	0	0	0	0	0	1	0	4
Radioactive waste	1	0	0	0	0	1	0	0	2	0	7	2	1
Pressurised containers	5	0	0	1	0	0	0	0	0	1	1	1	0

### Training/knowledge on health/environmental risks of HCWM, SHCWM practices and legislation on HCWM

#### Training

**Figure 2.**
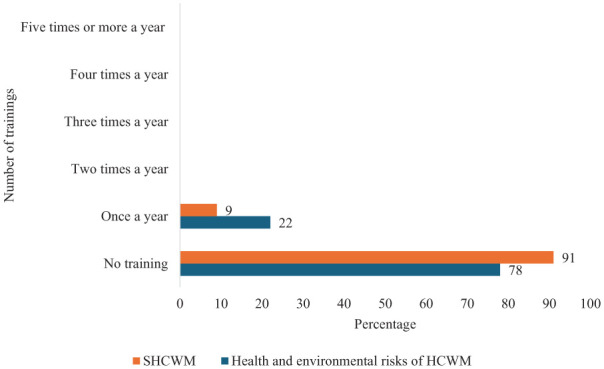
Training on health/environmental risks of HCWM and SHCWM. HCWM: healthcare waste management; SHCWM: sustainable healthcare waste management.

The respondents were asked to indicate how often they received ongoing training on health/environmental risks of HCWM and SHCWM practices such as waste prevention, minimisation, segregation, reuse and recycling. This did not include associated training as part of one-off staff induction programmes. As shown in Figure 2, majority of the respondents indicated they had no periodic training on either health/environmental risks of HCWM (78%) or SHCWM (91%). Only 22% and 9% said they had one training a year on health/environmental risks of HCWM and SHCWM, respectively. None of the respondents indicated they have had more than one training a year.

#### Knowledge

**Figure 3.**
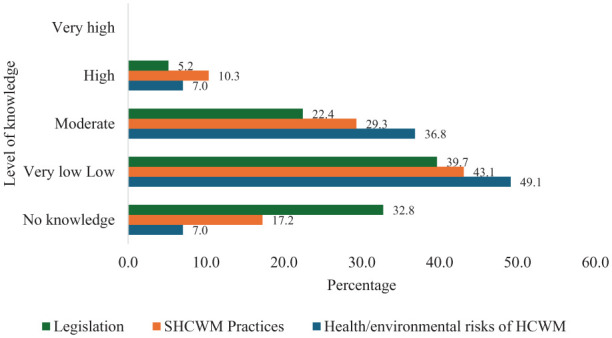
Knowledge on health/environmental risks of HCWM, SHCWM practices and legislation on HCWM in the United Kingdom. HCWM: healthcare waste management; SHCWM: sustainable healthcare waste management.

Regarding knowledge, the respondents were asked to rate their level of knowledge on health/environmental risks of HCWM, SHCWM practices and legislation on HCWM in the United Kingdom. As shown in Figure 3, only 6.9% indicated they had high knowledge of health/environmental risk of HCWM. About 36% indicated moderate knowledge, 48% had very low knowledge, and 7% said they had no knowledge.

Regarding SHCWM practices, only 10% indicated they had high knowledge of SHCWM practices. About 29% said they had moderate knowledge, 43% had very low knowledge, and 17% indicated they had no knowledge.

With regard to legislation, only 5% said they had high knowledge of HCWM legislation in the United Kingdom. About 22% said they had moderate knowledge, 40% had very low knowledge, and 33% saying they had no knowledge. The results show that only a small percentage of the respondents had high knowledge of health/environmental risks of HCWM, SHCWM practices and legislation on HCWM in the United Kingdom. Majority of the respondents had very low-to-moderate knowledge. None of the respondents indicated they had very high knowledge of either health/environmental risks of HCWM, SHCWM practices, or legislation on HCWM in the United Kingdom

### Evaluating perception, attitude and behaviour towards SHCWM

The respondents were asked a variety of questions ranging from their perception of HCW to attitudes on aspects of SHCWM, behaviour. The results in Table 4 show that only 76.8% disagreed with the perception that all waste generated within a healthcare facility is infectious. With regard to attitude, 89.7% said they were willing or strongly willing to attend voluntary programmes to update their knowledge about HCWM. Majority of the respondents 75.4% disagreed or strongly disagreed that segregation at source was extra work for them and time-consuming. Only 1.8% strongly agreed that segregation at source was extra work and time-consuming, whereas 17.5% agreed and 5.3% were undecided. Majority (81%) agreed or strongly agreed that they thought carefully before discarding waste items in available bins. With respect to whether segregation at source was beneficial, 93% agreed or strongly agreed that segregation at source improves recycle/reuse potential, whereas 82.8% either agreed or strongly agreed segregation at source reduced volume of hazardous/infectious waste. On whether SHCWM was beneficial to healthcare delivery, 72% indicated they believed SHCWM could enhance effective delivery of care at their Trust, whereas 93.1% indicated they believed it could enhance sustainability at their Trust and the healthcare sector. Regarding behaviour, 94.7% were willing to get involved with SHCWM initiatives, 98.3% willing to reuse materials if deemed safe. All respondents (100%) were willing to reuse devices and equipment if they were deemed safe. Majority (62.1%) indicated they had once put waste type in the wrong bin. For those who indicated to have put a waste type in the wrong bin, 58.3% indicated it was a rare occurrence, whereas 27.8% indicated it was not quite often, followed by 11% often. Only 2.8% said they did it all the time.

**Table 4.** Perception/attitude/behaviour towards SHCWM (*n* = 58).

Item				Response (%)	
Perception				Yes	No
All waste generated is infectious or hazardous				23.2	76.8
Attitude	Strongly agree	Agree	Not sure	Disagree	Strongly disagree
Segregation at source reduces volume of hazardous/infectious waste	43.1	39.7	15.5	1.7	
Segregation at source improves recycling/reuse potential	50.9	42.1	7		
I think carefully about the waste type before I discard waste items in the available bins	31	50	8.6	10.3	
Proper segregation of waste at source of generation is extra work for me and time consuming	1.8	17.5	5.3	49.1	26.3
SHCWM could enhance sustainability of my Trust and the health care sector	56.9	36.2	5.2	1.7	
SHCWM could enhance effective delivery of care my Trust and the health care sector	31.6	40.4	24.6	1.8	1.8
I am willing to attend voluntary programmes to update my knowledge about SHCWM practices	27.6	62.1	10.3		
Behaviour				Yes	No
Willingness to reuse devices and equipment if deemed safe				100	
Willingness to reuse materials if deemed safe				98.3	1.7
Willingness to get involved with SHCWM initiatives in my Trust				94.7	5.3
Have you ever put a waste type in the wrong bin?				62.1	37.9
		All the time	Often	Not quite often	Rarely
Use of wrong bin		2.8	11.1	27.8	58.3

SHCWM: sustainable healthcare waste management.

The demographic variables of age (18–34, 35–54, 55 and over), gender (male, female) and designation (doctors, nurses, clinical support, admin) were used to evaluate differences in means in perception, attitude and behaviour towards SHCWM as well as level of knowledge on health/environmental risks of HCWM, SHCWM practices and legislation on HCWM in the United Kingdom. A Kruskal–Wallis test showed a statistically significant difference in attitude across designation (χ² = 9.302, *df* = 4, α = 0.026, *p* < 0.05, *n* = 58). There were differences in means among doctors and clinical support staff (χ² = −15.408, standard error (SE) = 7.805, α = 0.048, *p* < 0.05, *n* = 58), nurses and administrative staff (χ² = −17.846, SE = 8.189, α = 0.029, *p* < 0.05, *n* = 58) and nurses and clinical support staff (χ² = −20.075, SE = 8.384, α = 0.017, *p* < 0.05, *n* = 58). Clinical support staff had higher mean scores (mean = 32.58), followed by administrative staff (mean = 30.00), then doctors (mean = 17.83) and lastly nurses (mean = 13.30), indicating that clinical support staff had generally more positive attitude towards SHCWM compared to nurses, doctors and admin staff. There was also statistically significant difference in attitude across age groups (χ² = 9.999, *df* = 4, α = 0.040, *p* < 0.05, *n* = 58). There were differences in means among those aged over 55 and 35–54 (χ² = 11.868, SE = 5.817, α = 0.041, *p* < 0.05, *n* = 58) and those aged over 55 and 18–34 (χ² = 20.269, SE = 6.870, α = 0.003, *p* < 0.05, *n* = 58). Those aged 18–34 had higher attitude mean scores (mean = 38.27), followed by 35–54 (mean = 29.87) and lastly over 55 (mean = 18.00), indicating that those under the age of 35 had generally more positive attitude towards SHCWM compared to those aged 35 and above. There was no statistically significant difference in perception across designation (χ² = 2.162, *df* = 3, α = 0.539, *p* > 0.05, *n* = 58) or age groups (χ² = 4.241, *df* = 4, α = 0.374, *p* > 0.05, *n* = 58), behaviour across designation (χ² = 1.995, *df* = 3, α = 0.573, *p* > 0.05, *n* = 58) or age groups (χ² = 5.438, *df* = 2, α = 0.066, *p* > 0.05, *n* = 58) and knowledge across age (χ² = 5.485, *df* = 2, α = 0.064, *p* > 0.05, *n* = 58) or designation (χ² = 3.071, *df* = 3, α = 0.381, *p* > 0.05, *n* = 58). Nurses had higher knowledge mean scores (mean = 37.70), followed by administrative staff (mean = 31.58), the doctors (mean = 28.92) and lastly clinical support staff (mean = 18.00). Those aged 35–54 had higher knowledge mean scores (mean = 38.27), followed by 18–34 (mean = 31.35), and lastly over 55 (mean = 18.95). A Mann–Whitney *U* test showed no statistically significant difference in attitude across gender (*U* = 340.000, σ = 61.375, α = 0.429, *p* > 0.05, *n*37, *n*21), behaviour across gender (*U* = 395.000, σ = 53.355, α = 0.903, *p* > 0.05, *n*37, *n*21) or knowledge across gender (*U* = 505.000, σ = 60.862, α = 0.056, *p* > 0.05, *n*37, *n*21). However, female had higher attitude mean scores (mean = 30.81) compared to males (mean = 27.19), whereas males had higher knowledge mean scores (mean = 35.05) compared to female (mean = 26.35), and marginally higher behaviour mean scores (mean = 29.81) compared to females (mean = 29.32).

## Discussion

### Training on health/environmental risks of HCWM and SHCWM

This study identified the lack of periodic training on health/environmental risks of HCWM and SHCWM. The findings revealed that overwhelming majority (78%) and (81%) did not receive periodic training on health/environmental risks of HCWM and SHCWM, respectively. This contrasts with Baaki et al. (2020) who reported that training was carried out up to four times a year in two observed teaching hospitals in Malaysia and that majority of respondents received training once a year on health/environmental risks of HCWM (46%) and SHCWM practices (41%). The findings are however similar in that marginally more received training on health/environmental risks of HCWM than they did on SHCWM practices. Training has often been cited as a significant factor in implementing safe and sustainable HCWM (Baaki, 2019; de Aguiar Hugo and Lima, 2021; Mathur et al., 2011; Ozder et al., 2013). The World Health Organization recommends routine training, at least annually and suggest such a training curriculum should cover areas such as injuries, infection prevention, potential hazards and use of PPE (Chartier et al., 2014). In this study, none of the respondents indicated they had received training more than once a year. Only 22% and 9% revealed they received training once a year on health/environmental risks of HCWM and SHCWM, respectively. This small percentage is suggestive of the lack of coordinated efforts to provide training on SHCWM across the observed NHS Trusts. The lack of training could also explain why some of the respondents chose wrong colour codes and likely put waste types in the wrong bin or receptacle. Botelho (2012) noted inadequate training and education as a major contributor to lack of compliance with relevant legislation on HCWM. Conti et al. (2024) on the other hand found that educational interventions were effective in improving knowledge, attitude and practices among healthcare workers. Training on risks of HCW and sustainable practices can be achieved through training programmes that include awareness campaigns such as instructive posters and signages, and regular formal training engagements.

### Knowledge on health/environmental risks of HCWM, SHCWM practices and legislation on HCWM in the United Kingdom

One of the objectives of this study was to evaluate staff knowledge on health/environmental risks of HCWM, SHCWM practices and legislation on HCWM in the United Kingdom. The findings revealed that majority of the respondents had very low to no knowledge on either health/environmental risks of HCWM (55%), SHCWM practices (70%) and legislation on HCWM in the United Kingdom (73%). Only 22% reported they had moderate knowledge on HCWM legislation in the United Kingdom, whereas only 29% indicated they had moderate knowledge on SHCWM practices. A higher number was reported for health/environmental risks of HCWM (36%). Considering the training reported in this study above, it is unsurprising to see that, only 10% of the respondents indicated they had high knowledge on health/environmental risks of HCWM, with less than 10% indicating they had high knowledge on SHCWM practices, and legislation on HCWM in the United Kingdom. These findings contrast with Baaki et al. (2020), Nazli et al. (2014) and Udayanga et al. (2023) who reported generally high knowledge from respondents on both health/environmental risks of HCWM and SHCWM practices. For example, Udayanga et al. (2023) reported high level of knowledge on HCWM and associated occupational health hazards among 76.9% of the respondents, whereas the study by Baaki et al. (2020) found that up to 76% and 78% had high to very high knowledge on health/environmental risks of HCWM and SHCWM practices such as waste prevention and minimisation, respectively. Although these findings also largely explain the findings on segregation practice in this study, it could also mean for some of the respondents that put waste types in the wrong bin, the reality could be they were only using the bins readily at their disposal. While the small percentage of hazardous HCW, if improperly handled such as using wrong bins could contaminate the entire HCW stream, exposing unsuspecting waste handlers to significant risks (Tudor et al., 2010), adding to treatment and disposal costs (Nichols et al., 2013) and limiting reuse/recycling potential. The study also found that overall knowledge on health/environmental risks of HCWM, SHCWM practices and legislation on HCWM in the United Kingdom was higher among nurses, 35- to 54-year-olds and females. Statistical tests showed no significant difference in mean knowledge among gender, age and designation. Similar findings were reported by Singh et al. (2022) who found there were no significant differences in knowledge among age, gender and designation of healthcare workers in a COVID-19 hospital in India.

### Perception, attitude and behaviour of staff towards SHCWM

Earlier findings in this study reported above indicate that training on a variety of HCWM aspects was inadequate, with majority of the respondents indicating very low to no knowledge on either health/environmental risks of HCWM, SHCWM practices or HCWM legislation in the United Kingdom. Regarding perception around HCW, 77% indicated they did not consider all waste generated to be infectious. Although this high number appear to reflect improvements in perception around HCW, it also reflects long standing negative perception of HCW due in part largely to reported failures in HCWM globally (Townend and Cheeseman, 2005). The findings on attitude indicated generally positive attitude towards SHCWM. Overwhelming majority of the respondents showed willingness to attend voluntary programmes to update their knowledge on SHCWM (90%). Regarding attitude towards segregation, 81% agreed they thought carefully before discarding waste items in bins, although 62% indicated to have at least once put waste in the wrong bin. It has been reported that, staff generally have a positive attitude towards segregation, however, not necessarily translated into practice. The respondents generally disagreed that segregation at source was extra work/time-consuming for them (75%). They agreed that segregation improved recycle/reuse potential (93%) and reduced volume of hazardous/infectious waste (83%). Most of the respondents believed that SHCWM could enhance effective delivery of care (72%) and overall sustainability of their Trust/healthcare sector (93%). The generally positive attitude is similar to findings by Lam et al. (2024) and Udayanga et al. (2023). With regard to behaviour, majority of the respondents indicated that, where deemed safe, they would reuse materials (98%), devices and equipment (100%). They also showed overwhelming willingness to get involved and participate in SCHWM initiatives (95%). A study by Tudor et al. (2007) in an NHS Trust in Cornwall found that staff generally reported thinking carefully about segregation; however, behaviour did not match intentions with large quantities of domestic-type HCW found in clinical waste bins. The statistical tests suggested that staff across different age groups and roles had different views and disposition towards SHCWM with statistically significant differences in overall attitude among age groups and designation. Those aged 18–34 years old had higher attitude mean scores suggesting those under 35 had generally more positive attitude towards SHCWM. This in similar to findings from a meta-analysis of literature by Wiernik et al. (2013) who found younger people generally showed more positive attitudes towards environmental sustainability.

## Conclusion

This study found deficiencies in training on key aspects of HCWM including health/environmental risks and sustainable practices. The study also found deficiencies in level of knowledge of hospital staff on risks of HCWM, sustainable practices and legislation on HCWM in the United Kingdom. There was largely no periodic training at the observed NHS Trusts and only a small number of staff reported training once a year. This lack of training reflected in knowledge levels and practice. Instances of using wrong bins to dispose waste were reported with majority having low to no knowledge. Although the findings demonstrated lack of periodic training and low levels of knowledge, there appeared to be a generally positive perception and attitude towards SHCWM practices. Where SHCWM initiatives are introduced, the findings suggest that staff would be willing to engage and participate. There were significant differences in perception and attitude across age ranges and staff in different roles. The key significant contribution of this study context to the existing inadequate training and knowledge on SHCWM by the NHS Trusts, likewise the sentiment around SHCWM practice post-COVID-19. This study therefore gives those leading sustainability efforts at NHS Trusts a snapshot of current sentiment towards SHCWM and elevates the need to develop periodic training programmes to improve staff knowledge and practice on SHCWM as part of efforts towards a net zero future. For more effective SHCWM implementation and uptake, policy at both Trust and national levels should incorporate mandatory minimum training exercises and awareness programmes, ensure allocation of adequate resources with clear mechanisms to assign accountability. For example, consideration should be given to mandating minimum periodic training exercises per year that aligns with specific roles of healthcare staff. These considerations by the policymakers will ensure compliance, growth in staff engagement in SHCWM, achieving overall sustainability goals. This study is not without limitations. The valid sample for this study was small despite repeated attempts to achieve higher participation and comprised majorly of respondents in non-clinical roles. It can be argued that the nature of waste they generated could likely have low or no hazardous components; therefore, the findings might not completely reflect the sentiment of those in clinical roles over a larger sample. Future studies could use a larger sample to explore knowledge, perception, attitude and behaviour across various regions within the United Kingdom to enable wider generalisation.
